# Electrophysiological and anatomical factors determine arrhythmic risk in acute myocardial ischaemia and its modulation by sodium current availability

**DOI:** 10.1098/rsfs.2019.0124

**Published:** 2020-12-11

**Authors:** Hector Martinez-Navarro, Xin Zhou, Alfonso Bueno-Orovio, Blanca Rodriguez

**Affiliations:** Department of Computer Science, British Heart Foundation Centre of Research Excellence, University of Oxford, Parks Road, Oxford OX1 3QD, UK

**Keywords:** myocardial ischaemia, coronary artery disease, anti-arrhythmic drugs, sodium block, Brugada syndrome, computer simulations

## Abstract

Acute myocardial ischaemia caused by coronary artery disease is one of the main causes of sudden cardiac death. Even though sodium current blockers are used as anti-arrhythmic drugs, decreased sodium current availability, also caused by mutations, has been shown to increase arrhythmic risk in ischaemic patients. The mechanisms are still unclear. Our goal is to exploit perfect control and data transparency of over 300 high-performance computing simulations to investigate arrhythmia mechanisms in acute myocardial ischaemia with variable sodium current availability. The human anatomically based torso-biventricular electrophysiological model used includes representation of realistic ventricular anatomy and fibre architecture, as well as ionic to electrocardiographic properties. Simulations show that reduced sodium current availability increased arrhythmic risk in acute regional ischaemia due to both electrophysiological (increased dispersion of refractoriness across the ischaemic border zone) and anatomical factors (conduction block from the thin right ventricle to thick left ventricle). The asymmetric ventricular anatomy caused high arrhythmic risk specifically for ectopic stimuli originating from the right ventricle and ventricular base. Increased sodium current availability was ineffective in reducing arrhythmic risk for septo-basal ectopic excitation. Human-based multiscale modelling and simulations reveal key electrophysiological and anatomical factors determining arrhythmic risk in acute ischaemia with variable sodium current availability.

## 1. Introduction

Acute myocardial ischaemia is a leading cause of sudden cardiac death, produced by reduced blood flow in the coronary arteries. Its causes include blood clot formation, atherosclerosis or vasospasm and its electrophysiological consequences increase the risk of lethal arrhythmias such as ventricular fibrillation. The risk is particularly high in the early phase of acute ischaemia (first 10–15 min post-occlusion) [1]. The occurrence of ischaemia-induced arrhythmias is also modulated by additional conditions, such as drug-induced effects and channelopathies. Thus, as reported in the clinical trial CAST (Cardiac Arrhythmia Suppression Trial), sodium channel blockers may increase arrhythmic risk in patients suffering from recurrent acute ischaemia episodes [2,3]. Furthermore, studies have highlighted the genetic predisposition to ischaemia-induced arrhythmias of patients with sodium current (*I*_Na_) channelopathies, such as Brugada syndrome or Lenègre's disease [4,5].

Huge advances in technologies and credibility of multiscale biophysical modelling and simulation have opened new avenues for the *in silico* evaluation of therapies and clinical conditions [6–10]. The multiscale nature of modelling and simulation allows unravelling underlying mechanisms from subcellular processes (such as ionic currents) to whole organ dynamics (such as reentrant arrhythmias) and the electrocardiogram. These techniques have been used to investigate the electrophysiological consequences of acute regional ischaemia on the ECG and arrhythmic risk, demonstrating excellent agreement with experimental and clinical recordings, as shown in [11–13]. Moreover, previous studies have shown the role of *I*_Na_ alterations caused by drugs and mutations in modulating arrhythmic risk in the human ventricles [14,15]. However, the interplay between acute ischaemia and *I*_Na_ availability in determining arrhythmia mechanisms is still unclear.

The aim of this study is to investigate the mechanisms of modulation of arrhythmic risk caused by altered *I*_Na_ availability in the human ventricles affected by the electrophysiological alterations caused by acute regional ischaemia. We hypothesize that both electrophysiological and anatomical factors determine the establishment of reentrant circuits in acute regional ischaemia and their modulation by *I*_Na_ availability.

## 2. Methods

### 2.1. Human torso-biventricular multiscale model of electrophysiology in acute regional ischaemia

The human biventricular model embedded in a torso, constructed and evaluated in our previous study [12] was used to simulate the concomitant effect of acute regional ischaemia and changes in sodium current availability from ionic dynamics to body surface potentials. In this study, the transmural ischaemia caused by LAD occlusion was selected in the simulations, since it was the most pro-arrhythmic case in [12]. The 12-lead ECG signal was computed from standard clinical lead positions on the torso. Details on the modelling and simulation framework and its validation are presented in [12].

In brief, human membrane kinetics were represented with the modified version of the O'Hara–Rudy (ORd) model [16] proposed by Dutta *et al*. [17] to overcome the limitations of the original model in reproducing electrical conduction and refractoriness under ischaemic conditions. Acute ischaemia was simulated in its early phase, before gap junctional uncoupling takes place, corresponding to high arrhythmic risk during the first 10–15 min post-occlusion, as indicated by experimental studies in animals [1,18]. Acquiring evidence on regional ischaemia-induced arrhythmia susceptibility in human would be challenging as most episodes occur out-of-hospital, and experimental ischaemia induction is constrained by ethical and also technical limitations (see also [19]). The ischaemic region was modelled as in [12,20], including (i) the ischaemic core zone (ICZ); (ii) the lateral border zone (BZ); and (iii) the endocardial BZ [21–23]. Ionic changes derived from the main electrophysiological effects of acute ischaemia (hyperkalemia, hypoxia and acidosis) were introduced in ischaemic tissue using values from experimental findings [21,23–25]. Hyperkalaemia led to an increased extracellular potassium concentration ([K^+^]_o_ = 9.5 mmol l^−1^); hypoxia enabled an ATP-dependent K^+^ current (*I*_K(ATP)_) by applying a scaling factor of 0.07 (0.00 if non-ischaemic) to the peak conductance of the current, estimated in 0.05 mS µF^−1^ [26]; and acidosis caused a decreased peak conductance of *I*_Na_ and L-type calcium currents (*I*_CaL_) by 25%. The BZ was modelled providing a linear transition in ischaemic parameters between the ICZ and the normal zone (NZ) tissue, as shown experimentally [22,23] and computationally [12,20,27–30]. NZ tissue had a [K^+^]_o_ of 5.4 mmol l^−1^ (baseline value in the ORd model), zero *I*_K(ATP)_ and default conductances for *I*_Na_ and the L-type calcium current as in the ORd model.

The bidomain equations were used to describe electrical conduction in the ventricular and torso domains and were discretized in space using a volumetric tetrahedral mesh, and solved with the finite-element method in CHASTE [31,32]. The spatial discretization of the myocardium was averagely 0.4 mm between nodes, which guaranteed numerical convergence [20,33]. The total volumetric mesh is based on 2.51 million nodes and 14.2 million tetrahedral elements.

Fibre orientation was imposed in the myocardial mesh with a rule-based method that reproduces the experimental findings by Streeter *et al*. [34]. Transmural and apico-basal electrophysiological heterogeneities were implemented in the myocardial model as described in [12] to reproduce experimental findings in healthy control conditions [35,36].

### 2.2. Stimulation protocols and quantification of the inducibility of arrhythmias

To simulate sinus rhythm, a realistic activation sequence [37] was implemented as described in [33], based on a fast conduction system mimicking propagation through the subendocardial Purkinje network at a propagation speed of 140 cm s^−1^. This yielded realistic QRS complexes in the 12-lead ECG. Four endocardial regular stimuli (S1) were applied with a cycle length of 1000 ms. Simulated ECGs and activation sequences were stabilized after the first two S1 stimuli, with the third and fourth stimuli producing consistent results. To evaluate reentry vulnerability, an additional ectopic stimulus (S2) was applied transmurally. This was based on the experimental evidence from [38] showing that the earliest activity of premature stimuli in acute myocardial ischaemia was identified in the normal myocardium adjacent to the ischaemic region, and that no important time differences were found between endo- and epicardium. The vulnerability window for reentry (VW) was defined as the range of coupling intervals (CI, i.e. time difference between the last S1 and S2) [6,28,39] which induced reentrant arrhythmias, as used previously for quantification of arrhythmic risk in whole-ventricular simulation studies [12,14,20]. Six different ectopic S2 locations were considered for each scenario, equally spaced around the ischemic BZ (in agreement with the location of ectopy in ischaemia experiments [22,38]). For each case, the simulated electrical activation pattern was analysed to identify the formation of reentrant circuits. In total, more than 400 simulations of 5–15 h each on 720 CPUs were conducted. Further details of the computational model of human ventricular electrophysiology in acute ischaemia can be found in [12].

### 2.3. Alterations in *I*_Na_ availability

In this study, we aim to analyse the effects of changes in *I*_Na_ availability in the formation of ischaemia-induced reentries. For this, we considered two scenarios with reduced *I*_Na_ availability. The first case involved a 50% reduction of the *I*_Na_ conductance with respect to baseline, which occurs in patients presenting SCN5A loss-of-function mutations [40]. The second scenario was a 25% reduction of the *I*_Na_ conductance with respect to baseline, which could represent drug-induced block. This degree of current block is estimated from a single pore block model [41], using the IC_50_ and *n*_H_ values for each drug–channel interaction extracted from [42]. We focused on the role of conductance in *I*_Na_ availability rather than current kinetics as considered in previous studies in healthy ventricles [15].

Clinical doses of sodium blockers vary (for example, the starting dose of flecainide is 100 mg per day, and the maximum recommended dose in 24 h is 600 mg [43,44]), resulting in variable drug concentrations in the patients' blood plasma. Table 1 shows the effects of the selected drugs on the *I*_Na_ at different doses: 1, 3 and 5 times the maximum effective free therapeutic plasma concentration EFTPC_max_. Taking into consideration dose variability and values shown in table 1, and especially flecainide, as related to the CAST, we considered 25% *I*_Na_ block to evaluate arrhythmic risk derived from a drug-induced *I*_Na_ blockage. Additionally, we also considered a 25% *I*_Na_ increase to evaluate the potential effect as proposed in [45]. These alterations in *I*_Na_ were implemented in the whole myocardium.

**Table 1.** *I*_Na_ block values computed for selected channel blockers at different doses: 1, 3 and 5 times the maximum effective free therapeutic plasma concentration EFTPC_max_. IC_50_ and *n*_H_ values for the different pharmacological compounds are extracted from [42].

drug	IC_50_ (nM)	*n*_H_	EFTPC_max_ (nM)	ionic block at EFTPC_max_ (%)	ionic block at 3× EFTPC_max_ (%)	ionic block at 5× EFTPC_max_ (%)
flecainide	6677	1.9	752.9	1.6	11.3	25.2
propafenone	3886	0.9	131.0	4.1	10.6	16.8
quinine	24 151	1.1	3956.7	11.8	31.2	44.5

### 2.4. Quantification of electrophysiological biomarkers

Due to the high computational cost of calculating effective refractoriness period (ERP) in different regions of the ventricle using the S1S2 simulation protocol, simulations of one-dimensional fibres were conducted to quantify electrophysiological characteristics with varying *I*_Na_ availability under control and ischaemic conditions in the absence of whole ventricle anatomical complexity. The fibres were 2 cm long with 100 nodes, and the stimulus current was applied to the first 3% of the fibres, and conductivity was set to 2.118 mS cm^−1^ to yield a realistic baseline longitudinal conduction velocity (CV) in humans. The one-dimensional fibres were paced under an S1–S2 protocol: regular S1 stimulus was applied with a cycle length of 1000 ms for 20 beats, and then an ectopic stimulus was set at progressively shorter CIs with 10 ms of decrement. ERP was defined as the shortest CI which enabled successful conduction at the end of the fibres. Action potential duration (APD) was calculated from the central node of the fibres from the last S1 beat as the time interval from depolarization to 90% of repolarization, and CV was measured from node 20 to node 80.

## 3. Results

### 3.1. Severe *I*_Na_ block led to moderate reduction in myocardial conduction velocity and significant increase in refractoriness dispersion

Table 2 shows how electrophysiological biomarkers vary with alterations in *I*_Na_ availability both in healthy and ischaemic tissue. Changes in *I*_Na_ availability altered mildly APD both under healthy and ischaemic conditions (first column). Refractoriness dispersion between healthy and ischaemic tissue was significantly increased under reduced *I*_Na_ availability (ΔERP under baseline conditions was 50 ms and with 50% *I*_Na_ block was 330 ms; see second column), which is associated with a higher likelihood of arrhythmias. Increased (25%) *I*_Na_ led to reduced refractoriness dispersion between the healthy and the ischaemic tissue (20 ms). *I*_Na_ block reduced myocardial propagation (longitudinal CV at baseline conditions was 70.6 cm s^−1^, whereas under 50% *I*_Na_ block conditions, longitudinal CV was 60 cm s^−1^). Under ischaemic conditions, the CV reduction is most pronounced in the case of *I*_Na_ block, leading to longitudinal CV values between 30 and 40 cm s^−1^, as shown in the third column. The resting membrane potential (*V*_rest_) was substantially increased by ischaemia (from −88 to −72.9 mV) but was not altered by variability in *I*_Na_ availability (last column).

**Table 2.** Measurement of electrophysiological properties under varying *I*_Na_ conditions, measured in one-dimensional fibres based on the O'Hara–Rudy model. APD and CV were measured under control and ischaemic conditions with the latter presented in parentheses. Refractoriness dispersion (ΔERP) was measured as the difference between refractory periods in ischaemic and healthy cells. One-dimensional fibres were stimulated with a frequency of 1 Hz.

scenario	APD (ms)	ΔERP (ms)	CV (cm s^−1^)	*V*_rest_ (mV)
baseline	240 (169)	50	70.6 (42.9)	−88.0 (−72.9)
50% *I*_Na_ block	239 (178)	330	60 (30.8)	−88.0 (−72.9)
25% *I*_Na_ block	241 (170)	140	66.7 (38.7)	−88.0 (−72.9)
25% *I*_Na_ increase	239 (167)	20	75 (48)	−88.0 (−72.8)

### 3.2. Different ectopic locations highlight the key role of ventricular anatomy in ischemia-induced reentrant mechanisms

**Figure 1.**
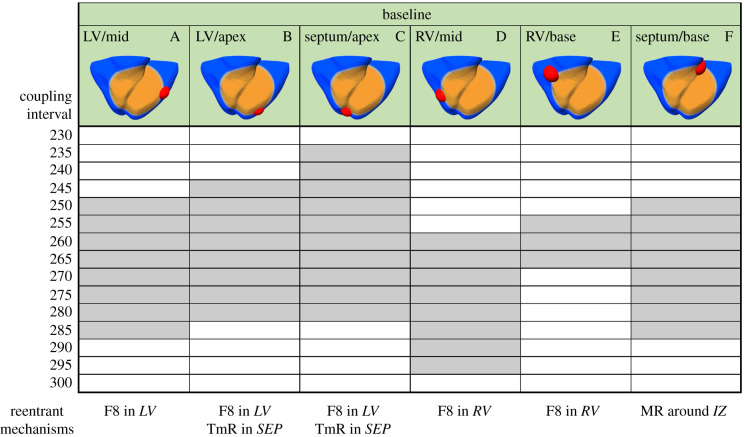
Vulnerability windows for reentry (grey boxes) for different locations of ectopic stimuli (red) in acute regional transmural ischaemia (orange) caused by LAD occlusion. Ectopic stimulation applied at CI = 230–300 ms at varying locations in the ischaemic border zone (cases A–F). Reentrant mechanisms observed in the vulnerability windows are annotated below. The reentry location (italic font) refers to the pathway for retrograde propagation. F8, figure-of-eight reentry; TmR, transmural micro-reentry; MR, macro-reentry; LV, left ventricle; SEP, septum; RV, right ventricle; IZ, ischaemic zone.

**Figure 2.**
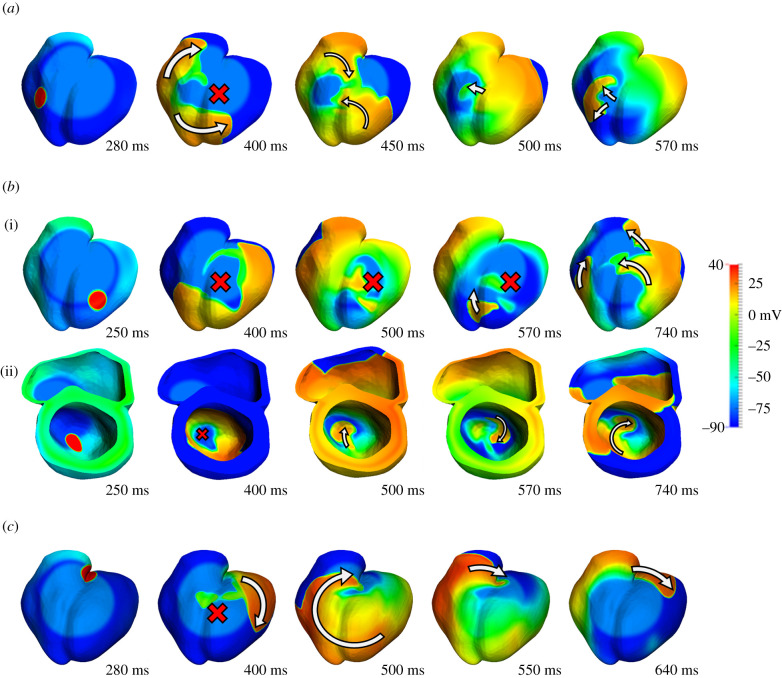
Main reentrant mechanisms observed in the ischaemia-induced reentry simulations. (*a*) Figure-of-eight macro-reentry, baseline ionic conditions, S2 triggered in the mid-RV at CI = 270 ms. (*b*) Transmural micro-reentry, baseline ionic conditions, S2 triggered in the LV/apex at CI = 250 ms. Epicardial (i) and septal view (ii). (*c*) Simple macro-reentry, baseline ionic conditions, S2 triggered in the septum/base at CI = 280 ms.

Figure 1 shows the VW obtained in the regionally ischaemic ventricles with baseline *I*_Na_ availability, for varying the location of the ectopic stimulus. The most pro-arrhythmic ectopic location was septum/apex (case C), followed by the left ventricle (LV) (cases A and B), right-ventricular (RV) mid-wall (case D) and septum/base (case F). The different ectopic locations also led to different types of reentry, as summarized in figure 1 (bottom panel) and illustrated in figure 2.

Ectopic stimulus in the LV (figure 1, cases A–C) and RV (figure 1, cases D and E) induced the establishment of figure-of-eight reentry, which is illustrated in figure 2*a*. Following the ectopic stimulus, unidirectional conduction block occurred in the ICZ, and propagation proceeded around the BZ (400 ms), then propagated retrogradely into the ICZ (450–500 ms), and reentered into the NZ of the RV (570 ms), as reported in [12,20,22].

In addition to figure-of-eight reentry, ectopic stimuli close to the apex (cases B and C) also led to the establishment of transmural micro-reentries (figure 2*b*). As shown in Martinez-Navarro *et al*. [12], the transmural reentry was established around the ischaemic BZ in the septo-apical region. Figure 2*b* shows that the ectopic stimulus caused a bidirectional conduction block (400–570 ms) and no reentry (740 ms) on the epicardium. Nevertheless, the ectopic stimulus propagated transmurally surrounding the BZ and reentered afterwards in the healthy tissue. This established a spiral wave, anchored in the BZ in the septo-apical region (figure 2*b*(ii), 500–740 ms).

Finally, ectopic stimuli close to the base were also pro-arrhythmic as shown by the wide VW (case F), due to the establishment of macro-reentry circling around the ICZ (figure 2*c*). This type of reentry occurred following the unidirectional block at the basal region of the RV, with late activation following regular stimulation. As the RV remained refractory from the previous regular beat, the wavefront propagated towards the apex through the LV, moved around the ICZ and then reached the excitable basal region, where the reentry was completed. In summary, the location of ectopic stimulus had crucial roles in ischaemia-induced arrhythmogenesis, leading to significant differences in the vulnerability to reentry and the patterns of reentrant pathways.

### 3.3. Severe reduction of *I*_Na_ availability promotes high inducibility of reentries following ectopy in right ventricle and septum/base

**Figure 3.**
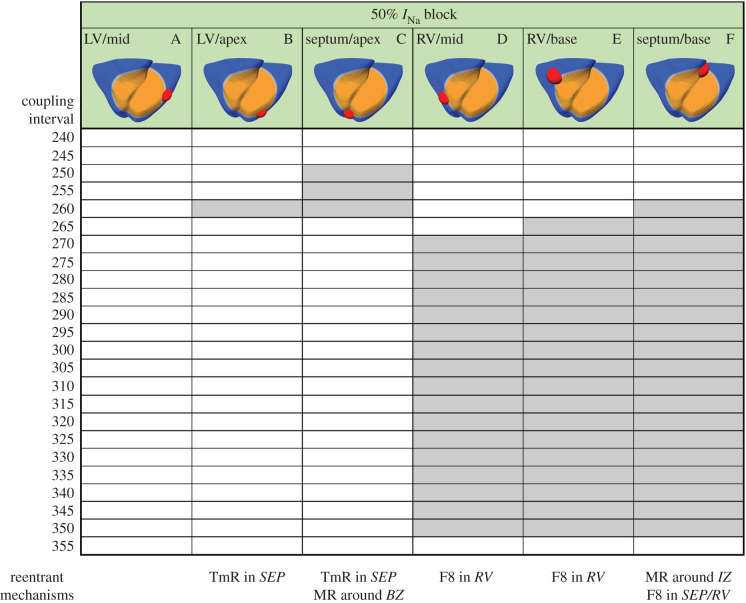
Vulnerability windows for reentry (grey boxes) for different locations of ectopic stimuli (red) in acute regional transmural ischaemia (orange) caused by LAD occlusion under 50% *I*_Na_ block. Ectopic stimulation applied at CI = 240–355 ms at varying locations in the ischaemic BZ (cases A–F). Reentrant mechanisms observed in the vulnerability windows are annotated below. The reentry location (italic font) refers to the pathway for retrograde propagation. F8, figure-of-eight reentry; TmR, transmural micro-reentry; MR, macro-reentry; LV, left ventricle; SEP, septum; RV, right ventricle; IZ, ischaemic zone.

Figure 3 shows the effect of 50% *I*_Na_ block on the VWs for the six ectopic locations considered. The results showed a large increase in the vulnerability for reentry for ectopic stimuli originated in the RV (figure 3, cases D and E) and at the base of the septum (figure 3, case F), whereas for ectopic stimulations in LV, the number of induced reentries decreased (figure 3, cases A–C). The most likely type of reentry was figure-of-eight with retrograde conduction through the LV towards the RV in cases D and E, and macro-reentry around the ICZ in case F. Ectopics in the LV and septum also led to some cases of transmural reentry (figure 3, cases B and C).

**Figure 4.**
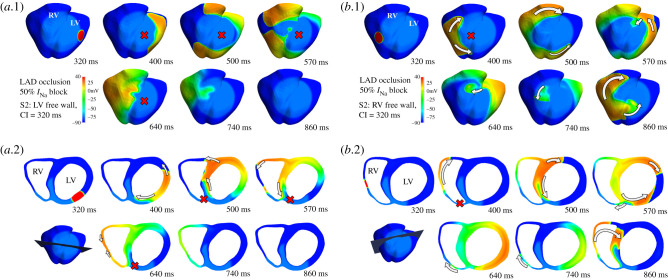
Comparison of activation sequences produced by ectopics set in LV and RV under 50% *I*_Na_ block. (*a*) Activation sequence triggered by an ectopic stimulus in the LV in simulated acute myocardial ischaemia caused by LAD occlusion with 50% *I*_Na_ block. (*a*.1) Epicardial view. (*a*.2) Slice visualization. No reentry was produced due to bidirectional conduction block in the anterior wall (represented by a red cross). (*b*) Activation sequence triggered by an ectopic stimulus in the RV in simulated acute myocardial ischaemia caused by LAD occlusion with 50% *I*_Na_ block. (*b*.1) Epicardial view. (*b*.2) Slice visualization. Unlike the stimulus originated in LV, the ectopic stimulus in the RV caused reentry based on unidirectional conduction block (400–500 ms, red cross), and posterior reentry to the RV (570–740 ms) leading to propagation towards septum and LV (860 ms).

Figure 4 illustrates the mechanism explaining the larger vulnerability to reentry for ectopic stimuli located in the RV versus the LV under the condition of 50% *I*_Na_ block by comparing two similar scenarios: ectopic set in LV (figure 4*a*) and RV (figure 4*b*) at similar distances in the long-axis plane and at a same coupling interval of 320 ms. Both in figure 4*a*,*b*, the top area shows an epicardial view and the bottom one a slice view of the short-axis plane, with changes in the orientation to optimally illustrate the different phases of reentry.

Figure 4*a*.1 shows that the ectopic stimulus in the LV free wall propagated around the BZ, due to a conduction block in the ICZ (400–500 ms). When the wavefront surrounded the ischaemic region (570 ms), retrograde propagation (640–740 ms) towards the LV died out, and reentry failed to be established (860 ms). Figure 4*a*.2 provides a transmural view, showing following ectopic stimulation, propagation surrounded the LV cavity via the LV free wall and also through the subendocardial BZ (400 ms). Then, it progressed through the RV free wall (500–570 ms) but eventually died out as both normal and ischemic tissue were refractory (640–740 ms). Unlike figure 4*a*.1, figure 4*b*.1 displays a complete macro-reentry, starting with the stimulus propagating around the BZ (400–500 ms).

Afterwards, a slow-propagating wavefront entered the ICZ through the septum, causing retrograde propagation towards the RV (570–740 ms). Finally, the reentry is completed and sustained (860 ms). From the transmural view in figure 4*b*.2, the ectopic stimulus propagated along the RV free wall due to the conduction block in the anterior ischaemic region (400 ms). Then, the wavefront propagated through the septum and LV through the inferior myocardial wall (500 ms). Depolarized tissue in the junction between the septum and LV wall eventually enabled a slow-propagating wavefront towards the thinner and excitable RV myocardial wall (570–640 ms). Once this wavefront propagated back into the healthy myocardium, the reentry was completed (740–860 ms).

**Figure 5.**
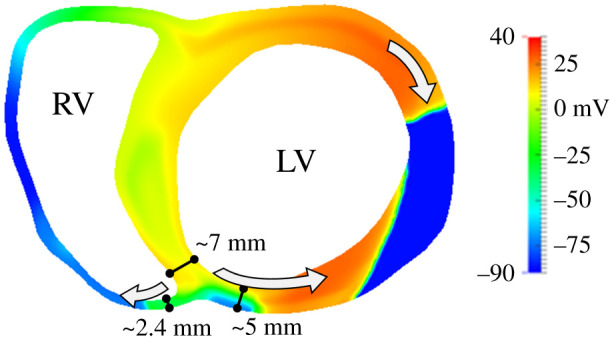
Quantification of myocardial wall width annotated over the activation maps at timestamp 570 ms from figure 4*b* (S2 in the RV). Note that the thick-to-thin transition promotes retrograde propagation towards the RV leading to the completion of the reentry (figure 4*b*, 860 ms).

The comparison between figure 4*a*,*b* proved the determining role of ventricular anatomy in modulating reentry establishment for different ectopic locations. The two ectopic S2 locations led to divergent outcomes under identical settings of ischaemic remodelling and sodium channel availability. At early stages, the activation sequence in both cases showed certain similarity (propagation of the stimulus, conduction block towards the septum and propagation across septum). The final stages were, however, very different: retrograde propagation was only possible from LV/septum to RV and not reversely. Figure 5 provides a quantification of the myocardial wall thickness in different regions. Myocardial wall thickness was measured close to the junction, with 7 and 5 mm in the septum and the LV, respectively. The RV wall was substantially thinner with a thickness of 2.4 mm, which established a substantial change in the propagation media that affected electrical conduction and promoted retrograde propagation only in the thick-to-thin direction. The LV/RV differences in wall thickness were particularly important under reduced *I*_Na_ conditions. Due to the remarkable increase in ΔERP induced, a major part of the ICZ remained refractory when the wavefront surrounded it. Moreover, propagation could proceed from the thick LV to the thin RV (figure 4*b*) but failed from RV to LV (figure 4*a*), due to the differences in source–sink mismatch, which were accentuated by the very low excitability present in scenarios with reduced *I*_Na_ and ischaemia.

### 3.4. Ectopic stimuli at the ventricular base are pro-arrhythmic for all degrees of *I*_Na_ availability tested

**Figure 6.**
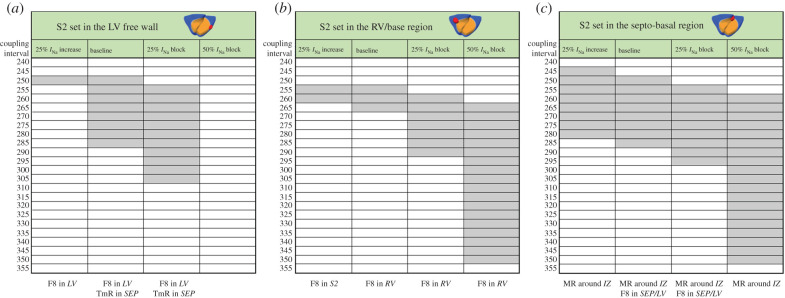
Vulnerability windows for reentry (grey boxes) for different locations of ectopic stimulus in acute regional transmural ischaemia caused by LAD occlusion with diverse *I*_Na_ availability. Ectopic stimulus applied at CI = 240–355 ms at three S2 locations: LV free wall (*a*), RV base (*b*) and septo-basal region (*c*). Reentrant mechanisms observed in the vulnerability windows are annotated (bottom). The reentry location (italic font) refers to the pathway for retrograde propagation. Each S2 location triggered certain types of reentry and varying *I*_Na_ availability affected the occurrence of these reentrant pathways. F8, figure-of-eight reentry; TmR, transmural micro-reentry; MR, macro-reentry; LV, left ventricle; SEP, septum; RV, right ventricle; IZ, ischaemic zone.

To further investigate the modulation of arrhythmic risk in ischaemia by *I*_Na_ availability, we conducted simulations considering 25% *I*_Na_ decrease and increase, to represent potential effects of *I*_Na_ block (table 1) as well as novel therapies as described in [45], respectively. Figure 6 shows VWs obtained with simulations with S2 located in the LV (figure 6*a*) and in the RV (figure 6*b*) at a similar distance from the ventricular base, and also in the septo-basal region (figure 6*c*) with progressively reduced *I*_Na_ availability for each stimulus location: 25% *I*_Na_ increase, baseline ionic conditions, 25% *I*_Na_ block and 50% *I*_Na_ block. The ectopic locations were chosen based on the high occurrence of reentries observed in the base and RV (figure 3, cases D and F), and to allow the comparison between RV and LV ectopy.

As shown in the previous section, 50% *I*_Na_ block (figure 6, right column in each VW table) caused very large VWs (85–95 ms), unless the S2 stimulus was located in the LV (VW = 0 ms), in which case the bidirectional conduction block hindered the formation of reentries, due to the differences in ventricular thickness between RV and LV, as explained above.

The 25% I_Na_ block promoted the formation of figure-of-eight macro-reentries caused by stimuli triggered in the LV or RV (figure 6*a,b*, third column), with the VWs 15–20 ms larger compared with baseline ischaemic conditions. However, if the ectopic stimulus was set in the base (figure 6*c*, third column), the VW was similar to that in baseline conditions (40–45 ms).

Previous research suggested that increasing *I*_Na_ availability [45] could be an effective anti-arrhythmic therapy, and our simulations also showed that 25% increase of *I*_Na_ reduced the electrophysiological ERP dispersion caused by ischaemia (table 2). For 25% increase in *I*_Na_ availability, VWs were smaller (LV: 5 ms, RV: 10 ms) than in baseline (LV: 40 ms, RV: 15 ms) for LV and RV ectopic locations (figure 6*a,b*, first column). Finally, ectopics at the base led to similar VW as in baseline conditions (40 ms), as illustrated in figure 6*c* (first column).

### 3.5. ECG changes caused by acute ischaemia and changes in *I*_Na_ availability

**Figure 7.**
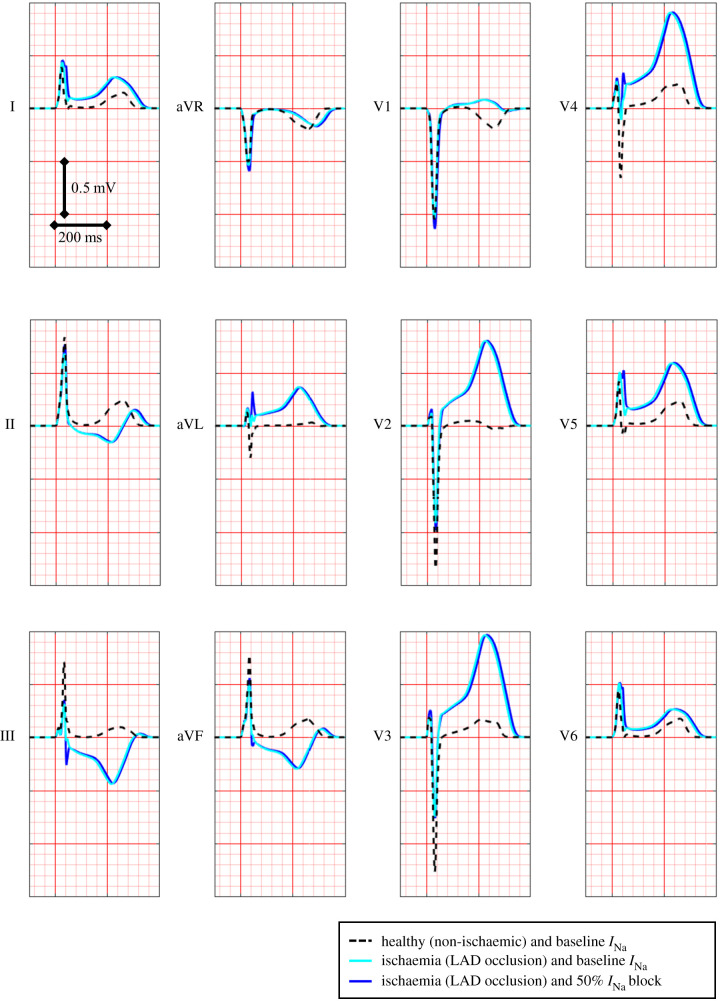
Simulated 12-lead ECG signal under control conditions (black dashed line), in ischaemia (light blue solid line) and ischaemia plus 50% *I*_Na_ block (dark blue solid line). Ischaemia leads to severe ECG changes, such as T-wave inversion in various leads (III, aVF and V1) and severe ST elevation in leads (V2–V4) in agreement with clinical data, as shown in [12]. ECG changes induced by additional 50% *I*_Na_ block in acute ischaemia are minor.

Figure 7 shows the simulated ECG computed for healthy control conditions and baseline *I*_Na_ (black dashed line) and transmural ischaemia from LAD occlusion, both in baseline *I*_Na_ conditions (light blue solid line) and 50% *I*_Na_ block (dark blue solid line). As evaluated in [12], ST elevation values obtained in simulated transmural ischaemia in baseline *I*_Na_ conditions (light blue solid line, 274–319 µV, leads V2, V3 and V4) are within the range obtained clinically during coronary balloon LAD occlusion (200 to 500 µV in leads V2, V3 and V4) [46]. Simulated 50% *I*_Na_ block in acute regional ischaemia (dark blue solid line) leads to minor ECG changes, including a very mild change in QRS morphology (III, aVL, V4) or width (I, V5, V6). The ECG changes do not reflect the changes in arrhythmic risk with altered *I*_Na_ current availability and the key role of ectopic location in its modulation explained in the previous sections.

## 4. Discussion

In our study, extensive human-based computer simulations reveal the important role of both electrophysiological and anatomical factors in the modulation of pro-arrhythmic reentrant activity by *I*_Na_ availability in acute regional ischaemia. For low *I*_Na_ availability, the asymmetry of the biventricular anatomy, and specifically the differences in thickness between RV and LV, play a critical role in explaining increased arrhythmic risk in acute ischaemia. These anatomical differences explain why ectopics originating from the RV and the basal region are very prone to cause reentry for reduced *I*_Na_ availability. Conversely, ectopic stimuli originating from the LV often fail to propagate from the thin RV back to the thick LV hindering the establishment of reentry. Furthermore, stimuli propagating from the basal region are highly arrhythmogenic, regardless of whether the *I*_Na_ is blocked or enhanced. The high arrhythmic risk induced by *I*_Na_ block did not correlate with the very minor changes it produced on the ECG. Thus, anatomical ventricular features as well as location of ectopic stimulus and ischaemic provide a more accurate prediction of arrhythmic risk than the analysis of ECG biomarkers.

Although sodium blockers are commonly used to treat certain cardiac arrhythmias, such as atrial fibrillation [43], their use is not recommended for patients under ischaemic risk, as evidenced by the CAST [47]. The results shown in this study provide explanations on the increased arrhythmic risk resulting from sodium block when coexisting with acute myocardial ischaemia. This aligns with the increased mortality observed in those CAST patients with recurrent acute ischaemia episodes produced by a reduced and uninterrupted coronary blood irrigation [2,3]. Previous studies have also shown increased arrhythmic risk in patients with reduced *I*_Na_ availability, especially under ischaemic conditions [4,5]. In addition to the electrophysiological abnormalities caused by sodium block (including slow conduction and increased dispersion of refractoriness), our simulations show that arrhythmogenesis under low *I*_Na_ availability was drastically influenced by the available anatomical pathways that cause reentry, which are linked to the location of the ectopic stimuli. The level of *I*_Na_ channel availability in the normal and ischaemic areas would be expected to vary significantly between individuals and ischaemic episodes. This would translate into differences in dispersion of refractoriness and arrhythmic risk, through the mechanisms unravelled in our simulations.

In simulations considering sodium block, ectopies in the RV were more prone to induce reentrant circuits than those in the LV due to differences in ventricular thickness and hence sink–source mismatch [48,49]. This is consistent with studies stating that conduction blocks are produced in the surface area with the steepest thin-to-thick transition [50,51]. Furthermore, a simulation study on non-ischaemic ventricles [14] identified source–sink mismatch under conditions of low *I*_Na_ availability as a potent substrate for sustained arrhythmia caused by ectopics triggered close to regions of wall expansion. In agreement with our simulations in ischaemic conditions, Boyle *et al.* identified the septum–RV intersection as the most prone region to cause conduction block, due to source–sink mismatch potentially leading to sustained reentries. Subsequently, the authors identified the right-ventricular outflow tract (RVOT) and the posteroinferior septal region of the RV as the most pro-arrhythmic ectopic locations. These results are fully consistent with our findings: (i) the simulations of low *I*_Na_ availability presented in this study identified a higher occurrence of reentries than in [14], which can be explained by the presence of acute myocardial ischaemia in the anterior myocardial wall; (ii) our results also identified the septal insertion region as crucial in the establishment of reentries; and (iii) although the ectopic locations in this study are not identical to those in Boyle's work, in both studies, the ectopics triggered in the basal and septal regions of the RV are the most prone to induce reentries.

In our study, stimuli originating from the septo-basal region caused the largest VWs, characterizing them not only as highly pro-arrhythmic, but also leading to robust reentrant circuits not affected by *I*_Na_ availability. These observations are consistent with previous clinical reports: the free-wall region of the RVOT has been identified as the most common origin of premature stimuli in Brugada patients [52], while Morita *et al*. [53] identified ectopics in that region as the most prone sites to induce ventricular fibrillation among Brugada patients. Note that in the reentries produced by septo-basal ectopic stimuli, retrograde propagation takes place from LV to RV, as seen in reentries caused by RV stimuli. Our simulation results demonstrated that the wider VWs caused by RV/basal ectopics were explained by mechanisms highlighting the interventricular differences in wall thickness as critical factors.

As discussed, severe *I*_Na_ block increased drastically arrhythmic risk under ischaemic conditions, in spite of very minor changes exhibited in the ECG (figure 7). Increased vulnerability to reentry could be explained by the very pronounced increase in dispersion in refractory period and yet moderate decrease in CV caused by *I*_Na_ block (table 2). However, *I*_Na_ block caused negligible ECG changes as previously reported in clinical studies, such as [54]. Our simulations highlight the fact that anatomical factors such as the location of the ischaemic region intersecting the antero-septal region [12] as well as the location of the premature ectopic stimulus in the RV or the base, as shown in this study, are critical factors for arrhythmic risk in acute ischaemia.

According to our simulations, increasing *I*_Na_ availability does not guarantee an effective anti-arrhythmic strategy for patients with unifocal ectopies originating from the basal region of the myocardium or adjacent area, as this reentrant pathway was relatively robust against the changes in *I*_Na_ availability. Increasing *I*_Na_ availability produced an anti-arrhythmic reduction in dispersion of refractoriness, mostly due to ERP shortening in the ischaemic tissue to values closer to the ones in the normal tissue. Therefore, its benefits very much rely on the intervention reaching the ischaemic tissue. However, short ERP in myocardial tissue allows a fast electrical recovery that provides instability to the myocardial activation sequence, as reported in [55–57]. Moreover, *I*_Na_ availability could be correlated to the occurrence of ectopic beats, as hypothesized in [47]. Therefore, a higher *I*_Na_ availability could lead to unwanted side effects, such as a higher occurrence of ectopic beats based on the increased excitability in the myocardial tissue. Our results suggest that increasing *I*_Na_ showed to be only partially effective as an anti-arrhythmic strategy for patients under ischaemic risk, given that the increase in *I*_Na_ could be ineffective in reducing ischaemia-induced arrhythmic risk for ectopic stimulation in the base. We propose our computational pipeline to investigate the safety and efficacy of other potential anti-arrhythmic strategies including therapy or drug-induced multi-channel block.

### 4.1. Limitations

The effects of sodium channel blockers are not limited to reducing the *I*_Na_ conductance, as it also affects the sodium channel kinetics [15]. Apart from reduced *I*_Na_ availability, Brugada syndrome patients suffer also heterogeneities in the RVOT (either in depolarization, repolarization or a combination of both) [58–60], or abnormal recovery from inactivation in specific SCN5A variants [61–63], to cite some, which were not considered in this study. However, decreased *I*_Na_ functionality is an important factor of these pathologies, and here we focused on isolating its modulation of arrhythmic risk under concurring acute regional ischaemia. Exploring the sensitivity to small variations in the S2 location represents a difficulty due to the high computational cost of the simulations.

## 5. Conclusion

Multiscale simulations using a human biventricular ventricular model show that anatomical factors as well as electrophysiological properties explain increased arrhythmic risk in acute ischaemia caused by changes in *I*_Na_ availability. Our results provide a mechanistic explanation for clinical studies identifying the RVOT as the most pro-arrhythmic ectopic location, for the high arrhythmic risk observed in patients with SCN5A mutations, and for life-threatening side effects of sodium blockers in ischaemic patients, as reported in the CAST. Under conditions of low *I*_Na_ availability, differences in RV versus LV thickness explain high arrhythmic risk in acute ischaemia, specifically for ectopic stimuli originating from the RV and ventricular base. The increased arrhythmic risk with low *I*_Na_ availability is not reflected in the ECG, which is hardly affected. Furthermore, increased *I*_Na_ availability was ineffective in reducing arrhythmic risk for septo-basal ectopics. The mechanisms unravelled through our simulations highlight the important role played by the asymmetric biventricular anatomy in modulating arrhythmic risk in acute regional ischaemia and its modulation by *I*_Na_ availability.

## Supplementary Material

Source code

## Data accessibility

The numerical solver CHASTE is freely available as open source at http://www.cs.ox.ac.uk/chaste/download.html. Meshes can be found at https://doi.org/10.5287/bodleian:9RxJPo9po; models and scripts to simulate simulation outputs are provided as electronic supplementary material.
